# Dosimetric Results for Adjuvant Proton Radiation Therapy of HPV-Associated Oropharynx Cancer

**DOI:** 10.14338/IJPT-D-21-00018

**Published:** 2021-11-24

**Authors:** Christopher M. Wright, Jonathan Baron, Daniel Y. Lee, Michele Kim, Andrew R. Barsky, Boon-Keng Kevin Teo, John N. Lukens, Samuel Swisher-McClure, Alexander Lin

**Affiliations:** Department of Radiation Oncology, University of Pennsylvania, Philadelphia, PA, USA

**Keywords:** Proton Therapy, Head and Neck Cancer, Oropharyngeal Cancer, Organs at Risk

## Abstract

**Purpose:**

One significant advantage of proton therapy is its ability to improve normal tissue sparing and toxicity mitigation, which is relevant in the treatment of oropharyngeal squamous cell carcinoma (OPSCC). Here, we report our institutional experience and dosimetric results with adjuvant proton radiation therapy (PRT) versus intensity-modulated radiotherapy (IMRT) for Human Papilloma Virus (HPV)-associated OPSCC.

**Materials and Methods:**

This was a retrospective, single institutional study of all patients treated with adjuvant PRT for HPV-associated OPSCC from 2015 to 2019. Each patient had a treatment-approved equivalent IMRT plan to serve as a reference. Endpoints included dosimetric outcomes to the organs at risk (OARs), local regional control (LRC), progression-free survival (PFS), and overall survival (OS). Descriptive statistics, a 2-tailed paired *t* test for dosimetric comparisons, and the Kaplan-Meier method for disease outcomes were used.

**Results:**

Fifty-three patients were identified. Doses delivered to OARs compared favorably for PRT versus IMRT, particularly for the pharyngeal constrictors, esophagus, larynx, oral cavity, and submandibular and parotid glands. The achieved normal tissue sparing did not negatively impact disease outcomes, with 2-year LRC, PFS, and OS of 97.0%, 90.3%, and 97.5%, respectively.

**Conclusion:**

Our study suggests that meaningful normal tissue sparing in the postoperative setting is achievable with PRT, without impacting disease outcomes.

## Introduction

Oropharyngeal squamous cell carcinoma (OPSCC) is an increasingly diagnosed cancer, with more than 53 000 annual cases in the United States [1]. It is largely driven by increasing rates of human papillomavirus (HPV) infection [2] and generally associated with highly favorable prognosis [3–6] after treatment with either definitive chemoradiation or surgery followed by adjuvant radiation therapy (with or without chemotherapy, as indicated) [7].

Even the use of the most advanced forms of standard radiation therapy, such as intensity-modulated radiation therapy (IMRT), results in significant toxicity [8], negatively impacting long-term quality of life [9, 10]. Proton therapy (PRT), with its unique physical characteristics (Bragg peak) and ability to potentially improve normal tissue sparing, can have a significant role in toxicity mitigation. Early reports of proton therapy suggest a favorable toxicity profile as compared to IMRT, likely owing to the improved sparing of organs at risk (OARs) [11–13], with reductions in xerostomia, dysgeusia, feeding-tube dependency, and severe weight loss [14, 15], and improvement in patient-reported outcomes [16].

Given the promise of PRT and its current use in randomized controlled trials (NCT01893307 and ISRCTN16424014) [17, 18], there is a need to understand what dosimetric results are obtainable with proton therapy for normal tissues and organs at risk. Our study, which is the largest to date analyzing postoperative PRT for OPSCC, reports the following: (1) achievement of dosimetric values for critical OARs (without compromising excellent disease outcomes), and (2) the superiority of these values as compared with treatment-approved IMRT plans. In so doing, we hope that this can lead, through current and future efforts, to improved patient outcomes through toxicity mitigation, and to help inform future prospective efforts in which proton therapy will be used.

## Materials and Methods

### Patients

This is an institutional review board-approved study of patients with HPV-associated OPSCC, treated at the University of Pennsylvania (between 2015 and 2019) initially with transoral robotic surgery for resection of the primary tumor and selective neck dissection, followed by adjuvant proton radiation, with or without chemotherapy (according to standard indications) [19].

### Treatment Planning and Dosimetric Computations

All patients were treated with standard postoperative doses (60-63 Gy in 30 fractions, using a conversion factor of 1.1 relative biological effectiveness [RBE]), including the primary tumor operative bed and bilateral neck, with delineation of OARs and target echelon nodal regions, based on published and commonly used guidelines [20–23]. Patients with close margins (<2 mm) or extranodal extension were treated to 63 Gy in 30 fractions. Treatment planning for PRT was performed via Eclipse (version 11, 13, or 15 [depending on year], Varian Medical Systems, Palo Alto, California), with planning and delivery via pencil beam scanning. Plans prior to 2017 used the single-field optimization (SFO) technique (2015-2017), while newer plans used the multi-field optimization (MFO) (2017-2019) technique when robust optimization was clinically available. A 3-beam configuration consisting of 2 posterior oblique fields was used to cover the superior portion of the CTVs, while 1 anterior field was used to cover the inferior portion of the CTVs [24]. In the SFO technique, the plan was optimized to cover the CTVs plus a uniform 5-mm margin to account for setup uncertainty and proton-range uncertainty in the beam direction. The MFO technique used robust optimization parameters of 3 mm for setup and 3.5% for proton-range uncertainty. Clinically acceptable SFO plans required 95% of the target volume (CTV + 5 mm) to receive at least 95% of the prescribed dose, while MFO plans were evaluated with the worst-case uncertainty scenario of 95% CTV volume to receive at least 95% of the prescribed dose. For either technique, hotspot defined as D0.03 cm^3^ was limited to ≤110% of the prescribed dose.

Dosimetric data for all structures was extracted from Eclipse using dose-volume histogram data obtained from the PRT plans and treatment-approved IMRT plans generated using initial computed tomography (CT) simulation imaging data and physician-delineated volumes.

### Statistical Analysis

Descriptive statistics were used to characterize the baseline characteristics of the overall population. A 2-tailed paired *t* test was used to compare dosimetric outcomes between the PRT and IMRT plans. Survival times were computed from radiation therapy end date to the occurrence of the first event. Events were death from any cause for overall survival (OS) and any recurrence or death for progression-free survival (PFS). Local and/or regional recurrences were deemed as events for local regional control (LRC) analysis. Survival rates were estimated by using the Kaplan-Meier method; patients were censored after a recorded event or last follow-up visit. Statistical analyses were performed using Stata SE, version 15.0 (StataCorp, College Station, Texas).

## Results

A total of 53 patients were identified who met the study criteria and underwent standard dose and volume postoperative PRT from 2015 to 2019 (Table 1) [25]. The median follow-up time for the cohort was 20.4 months (range, 6-50 months).

**Table 1.** Patient characteristics.

**Patient characteristics**	**Value, n (%), N = 53**
Age at RT, median (range)	62 (47-77)
AJCC TNM pathologic stage [xx]	
Stage I	34 (64)
Stage II	19 (36)
Stage III/IV	0
Pathologic T stage	
pT0	3 (6)
pT1	24 (45)
pT2	16 (30)
pT3	6 (11)
pT4	4 (8)
Pathologic N stage	
pN0	2 (4)
pN1	41 (77)
pN2	10 (19)
Clinical M stage	
cM0	53 (100)
cM1	0
Primary tumor site	
Tonsil	26 (49)
Base of tongue	19 (36)
Other	8 (15)
Concurrent chemotherapy	
None	37 (70)
Cisplatin	15 (28)
Cetuximab	1 (2)
Year of RT	
2019	6 (11)
2018	23 (43)
2017	9 (17)
2016	10 (19)
2015	5 (9)
RT dose	
60 Gy	31 (58)
63 Gy	22 (42)

**Abbreviations:** AJCC, American Joint Committee on Cancer; TNM, tumor, node, metastasis; RT, radiation therapy.

**Figure 1.**
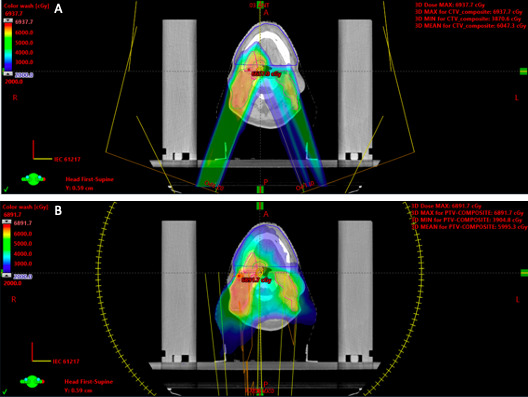
Sparing of the oral cavity with proton therapy versus IMRT rapid arc. Seventy-year-old male with a stage I (pT1pN1cM0) HPV-associated right palatine tonsil squamous cell carcinoma with extranodal extension noted on surgical pathology. The patient underwent adjuvant chemoradiotherapy to 63 Gy in 30 fractions with PRT. (A) PRT allowed for improved sparing of the oral cavity (blue) compared with (B) the treatment-approved rapid arc plan. Mean dose to the oral cavity was 8.5 Gy and 23.5 Gy for PRT and rapid arc plans, respectively. Abbreviations: IMRT, intensity-modulated radiotherapy; PRT, proton radiation therapy.

The significant reductions in dose to the analyzed OARs achieved with PRT as compared with treatment-approved IMRT are demonstrated in Table 2. Apparent gains with proton therapy were most notable for the pharyngeal constrictors, esophagus, larynx, oral cavity, and contralateral salivary glands (parotid and submandibular). A representative proton radiation plan compared with IMRT is shown in Figure 1, demonstrating significant sparing of the oral cavity.

**Table 2.** Normal tissue sparing achieved with proton therapy compared with IMRT.

**Organ at risk**	**PRT: Mean dose (95% CI), Gy**	**IMRT: Mean dose (95% CI), Gy**	***P*** **value**
Constrictors	29.6 (27.5-31.6)	37.3 (36.0-38.6)	<.00001
Esophagus	10.5 (8.9-12.1)	15.6 (14.2-17.1)	<.00001
Larynx	21.6 (19.9-23.3)	27.4 (25.6-29.1)	<.00001
Mandible maximum	60.5 (59.4-61.6)	62.6 (61.6-63.6)	<.00001
Oral Cavity	5.0 (4.0-6.1)	20.5 (19.2-21.7)	<.00001
Ipsilateral parotid	24.8 (23.5-26.1)	30.8 (21.2-40.5)	.0211
Contralateral Parotid	12.2 (10.9-13.5)	18.1 (16.8-19.3)	<.00001
Spinal Cord maximum	36.5 (34.8-38.2)	39.0 (37.7-40.3)	.00129
Contralateral Submandibular	28.7 (26.9-30.5)	31.0 (28.9-33.1)	.00002

**Abbreviations:** PRT, proton radiation therapy; CI, confidence interval; IMRT, intensity-modulated radiotherapy.

**Figure 2.**
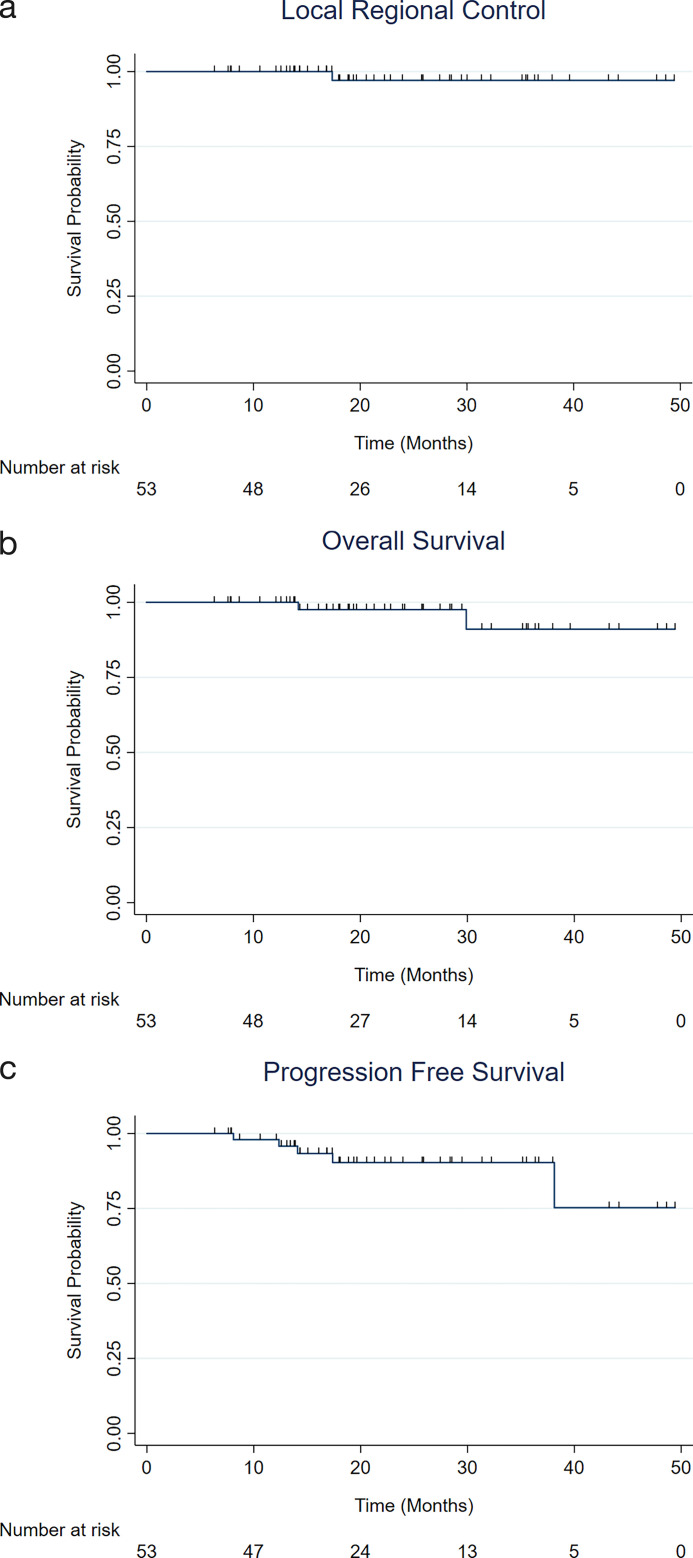
Disease-specific outcomes: (A) local regional control, (B) progression-free survival, and (C) overall survival. Kaplan-Meier estimates of (A) local reg control, (B) progression-free survival, and (C) overall survival for the overall patient cohort. The median follow-up time was 20.4 months (range, 6-55 months).

Our achieved dose sparing did not compromise disease outcomes, with only 1 observed local regional recurrence (0 local, 1 regional). Two-year LRC, PFS, and OS were 97.0% (95% CI, 80.9%-99.6%), 90.3% (75.9%-96.3%), and 97.5% (83.9%-99.7%), respectively (Figure 2).

## Discussion

Our study of patients who received adjuvant PRT for HPV-associated OPSCC demonstrates improvements in the dosimetric sparing of critical OARs as compared with both treatment-approved IMRT plans. Significant reductions in dose to the constrictors, esophagus, oral cavity, larynx, and contralateral salivary glands were achieved. These improvements in normal tissue sparing did not appear to adversely affect disease outcomes. Our study showed very high rates of local regional control (97%) as compared with previously published results from a similar patient population [26] and treated to a comparable dose [27, 28]. Our findings contribute to the existing literature of PRT for OPSCC. Uniquely, our study homogeneously focuses on a previously understudied patient population consisting solely of postoperative HPV-associated OPSCC requiring adjuvant radiation therapy. Ultimately, our findings suggest that significant dose reductions can be consistently achieved with proton therapy in a population for whom toxicity mitigation to improve posttreatment quality of life is of vital importance. Furthermore, the dose sparing that we achieved can be used to inform future trials of proton therapy in this patient population.

The findings demonstrated in our study are consistent with and build upon prior reports of PRT for OPSCC. A previous review of 25 patients with intact head and neck cancers (21 patients with OPSCC) showed significant reductions in dose to the ipsilateral parotid gland and supraglottic larynx with PRT compared with IMRT [11]. Similarly, a report of 25 patients with OPSCC treated with definitive chemoradiation at MD Anderson showed reductions in the mean doses to the oral cavity, hard palate, larynx, mandible, and esophagus with PRT compared with IMRT [13]. Our study is the largest of its kind for patients receiving bilateral neck proton radiation therapy for oropharynx cancer in the postoperative setting.

## Conclusion

There are several limitations to our study. First, our data are limited to a homogeneous population of patients receiving postoperative proton therapy after surgical resection for oropharyngeal cancer at a single institution. Treatment techniques may differ between institutions and impact the dose to the studied OARs. For example, we use a combined posterior oblique and anterior beam approach, whereas other centers use anterior obliques and a posterior beam approach [13]. Second, current trials in oropharynx cancer are examining the use of lower therapeutic doses than received by our patients; however, even if standard of care evolves in the future toward lower than current doses, we believe that our findings showing PRT as superior to IMRT for dosimetric sparing will not change. Finally, our data were limited to dosimetric data and do not include measures focusing on patient toxicity or quality of life; such questions will be important to address in future prospective trials of proton therapy, to ensure the best possible outcomes for patients.

In summary, we report notable normal tissue sparing with proton therapy in the postoperative setting for oropharynx cancer. We recommend and plan for a longer follow-up period and evaluation of a larger number of patients in the future to confirm and ensure that our treatment approach with proton therapy will ultimately lead to the best outcomes for patients.
